# Highlighting the Relevance of Gut Microbiota Manipulation in Inflammatory Bowel Disease

**DOI:** 10.3390/diagnostics11061090

**Published:** 2021-06-15

**Authors:** Flavia Maria Pavel, Cosmin Mihai Vesa, Gina Gheorghe, Camelia C. Diaconu, Manuela Stoicescu, Mihai Alexandru Munteanu, Elena Emilia Babes, Delia Mirela Tit, Mirela Marioara Toma, Simona Bungau

**Affiliations:** 1 Department of Preclinical Disciplines, Faculty of Medicine and Pharmacy, University of Oradea, 410073 Oradea, Romania; flavia.bontze@gmail.com (F.M.P.); v_cosmin_15@yahoo.com (C.M.V.); 2 Department 5, “Carol Davila” University of Medicine and Pharmacy, 050474 Bucharest, Romania; gheorghe_gina2000@yahoo.com (G.G.); drcameliadiaconu@gmail.com (C.C.D.); 3 Department of Internal Medicine, Clinical Emergency Hospital of Bucharest, 105402 Bucharest, Romania; 4 Department of Medical Disciplines, Faculty of Medicine and Pharmacy, University of Oradea, 410041 Oradea, Romania; manuela_stoicescu@yahoo.com (M.S.); mihaimunteanual@yahoo.com (M.A.M.); eebabes@uoradea.ro (E.E.B.); 5 Department of Pharmacy, Faculty of Medicine and Pharmacy, University of Oradea, 410028 Oradea, Romania; mirela_tit@yahoo.com (D.M.T.); mire.toma@yahoo.com (M.M.T.); 6 Doctoral School of Biological and Biomedical Sciences, University of Oradea, 410087 Oradea, Romania

**Keywords:** gut microbiota, supplements, inflammatory bowel disease, Crohn’s disease, ulcerative colitis, probiotics, prebiotics, fecal microbiota transplant, phytochemicals

## Abstract

Two different conditions are included in inflammatory bowel disease (IBD), Crohn’s disease (CD) and ulcerative colitis (UC), being distinguished by chronic recurrence of gut inflammation in persons that are genetically predisposed and subjected to environmental causative factors. The normal structure of the gut microbiome and its alterations in IBD were defined in several microbial studies. An important factor in the prolonged inflammatory process in IBD is the impaired microbiome or “dysbiosis”. Thus, gut microbiome management is likely to be an objective in IBD treatment. In this review, we analyzed the existing data regarding the pathophysiological/therapeutic implications of intestinal microflora in the development and evolution of IBD. Furthermore, the main effects generated by the administration of probiotics, prebiotics, fecal transplantation, and phytochemicals supplementation were analyzed regarding their potential roles in improving the clinical and biochemical status of patients suffering from Crohn’s disease (CD) and ulcerative colitis (UC), and are depicted in the sections/subsections of the present paper. Data from the literature give evidence in support of probiotic and prebiotic therapy, showing effects such as improving remission rate, improving macroscopic and microscopic aspects of IBD, reducing the pro-inflammatory cytokines and interleukins, and improving the disease activity index. Therefore, the additional benefits of these therapies should not be ignored as adjuvants to medical therapy.

## 1. Introduction

Inflammatory bowel diseases (IBD) are chronic, immune-mediated conditions, which affect the gastrointestinal tract. The term IBD includes Crohn’s disease (CD) and ulcerative colitis (UC), which both present an elaborate etiology and pathogenesis that has been insufficiently described and acknowledged. IBD occurrence has become more frequent over time, this being connected to industrial progress and innovation and related lifestyle changes. The prevalence of these diseases is higher in developed countries, where it can reach up to 4 cases per 1000 inhabitants [1]. From an epidemiological point of view, the incidence of IBD varies by two criteria, that is, geographical region and age. Thus, in the North American region, the incidence of UC varies between 2.2 and 19.2 cases per 100,000 inhabitants, and the incidence of CD between 3.1 and 20.2 per 200,000 inhabitants [2]. According to age, there is a bimodal distribution of IBD, with a first peak between 15 and 30 years, and the second peak after 60 years [2]. If approximately 25% of patients develop IBD in adolescence, 10–15% of them may have IBD onset after the age of 60 years [2].

Currently, the exact pathophysiological mechanism leading to IBD is not known, one of the hypotheses being the existence of an aggressive immune response to the intestinal microbiota in genetically predisposed individuals. This hypothesis is supported by recent studies that have identified susceptibility loci at or near genes involved in the innate or adaptive immune response to various germs [2,3,4,5]. However, there are numerous studies aimed at identifying non-immunosuppressive therapeutic agents with a possible benefit for patients with these conditions. These agents target microbial flora disorders that accompany IBD and include prebiotics, probiotics, antibiotics, or fecal microbial transplantation. Unfortunately, these studies have failed to demonstrate a definite benefit of drugs targeting the intestinal microflora and, as a result, have not been approved in current therapeutic guidelines.

The main two entities that are part of IBD, CD and UC, are differentiated by the location of the lesions and the layers of the digestive tract wall involved. Thus, CD is characterized by the involvement of all the digestive tract layers, otherwise known as transmural damage, which can induce inflammation, strictures, or even fistulas. In the advanced stages, the mucosa acquires the appearance of cobblestone by the presence of linear ulcerations alternating with areas of normal mucosa [2,3,4]. CD lesions can be present in any segment of the digestive tract. The ileum and colon are most frequently affected, the gastroduodenal segments being involved in only 5% of cases [4]. The upper gastrointestinal tract may be more commonly involved in childhood CD forms [4]. Typically, the rectum is spared in CD, but anorectal complications (fistulas and abscesses) are common [2,4]. UC, unlike CD, is characterized by diffuse inflammation in the colonic mucosa, most commonly affecting the rectum (proctitis). However, UC lesions may extend to the sigmoid (proctosigmoiditis), beyond the sigmoid (distal ulcerative colitis), or may include the entire colon (pancolitis) [2]. The two diseases can be classified depending on the extension into mild, moderate, and severe forms, and depending on the location [2]. Additionally, for CD, there is a phenotypic classification into inflammatory, structuring, or penetrating forms [2]. Both patients with CD and those with UC can associate extraintestinal manifestations that may involve the eyes, skin, and bones, such as arthritis, ankylosing spondyloarthropathy, uveitis, aphthous stomatitis, or erythema nodosum [4].

This article centralizes the existing data provided by significant literature published between 1989 and 2021 and related to the pathophysiological and therapeutic implications of intestinal microflora in the development and evolution of IBD; moreover, it presents in detail the beneficial effects of probiotics, prebiotics, phytochemicals supplementation, or fecal transplant in improving the clinical parameters of IBD patients. In this regard, the authors searched the most well-known databases (i.e., MDPI, Scopus, ScienceDirect, Elsevier, Frontiers, etc.), using key words or combinations of them (i.e., gut microbiota; supplements; inflammatory bowel disease; Crohn’s disease; ulcerative colitis; probiotics; prebiotics; fecal microbiota transplant; phytochemicals, etc.). As a result, 172 references were cited as supporting the statements in this work.

Moreover, the present research intends to provide specialists and patients with new information related to this topic and to make as accessible as possible the published data regarding the newest/latest therapies in the field; in addition, the most pertinent and relevant results obtained in medical practice were registered, focused on the optimization of the management of this pathology, both by examining the valuable scientific evidences and by presenting/respectively evaluating the modern ways of treating this disease.

## 2. Gut Microbiota in the Pathogenesis of IBD

There are currently numerous studies that have investigated the relationship between intestinal microflora and IBD [3]. These studies have demonstrated the essential role that the microbiome plays in the development and evolution of intestinal inflammation [3,6]. According to existing data already published, the intestinal microflora of patients with IBD present an increase in the number of bacteria from the *Proteobacteria phylum* family and a decrease in those from the *Firmicutes phylum* and Bacterioides families, compared to normal individuals [4,7,8,9,10]. In addition, the diversity of bacterial microflora, known as α diversity, is lower in patients with intestinal inflammation [4,11,12]. Patients with CD show a reduction in α diversity in fecal microbiomes, a change also identified among monozygotic twins [11,12]. This dysbiosis has been associated with the instability of microbial species dominance in IBD [13]. There are data that show a reduction in microbial diversity in tissues with inflammatory changes, compared to those without inflammatory changes, even in the same patient [14]. A similar dysbiosis was observed in patients with colitis secondary to *Clostridium difficile* infection, suggesting the possibility of using treatments for this infectious disease in patients with IBD [4,15].

A multicenter study that looked at more than 1000 fecal samples from children with CD has found an increase in the number of species from the Veillonellaceae, Pasteurellacaea, and Enterobacteriaceae families, and a decrease in those from the Bacteroidales, Erysipelotrichales, and Clostridioides families [16]. These changes have also been associated with disease status [16]. Another important observation of this study is the possibility of using the microbial profile of the rectal mucosa as a biomarker for the diagnosis of CD in its early stages [16].

From the patho-physiological point of view, the association of intestinal dysbiosis with the appearance of inflammatory changes can be explained by the increase in the number of bacteria with a proinflammatory role and the reduction of those with an anti-inflammatory role, compared to healthy individuals [9,10]. For example, *Faecalibacterium prausnitzii,* which is part of the Clostridium IV family, has demonstrated an anti-inflammatory role due to its production of butyrate. In patients with CD, there is a reduction in the number of bacteria from the *Faecalibacterium prausnitzii* family, a reduction that has been correlated with the risk of relapse after surgical treatment among these patients [17,18].

Additionally, a reduction in the number of *Blautiafaecis*, *Roseburiainulinivorans*, *Ruminococcus torques*, and *Clostridium lavalense* has been demonstrated in patients with CD [17,18]. In UC, during the remission period, changes in the colonization of *Faecalibacterium prausnitzii* were observed, and the restoration of the population of this bacterial species after relapse is associated with the maintenance of clinical remission. An important role of *Faecalibacterium prausnitzii* has been demonstrated by Sokol et al. They showed that the stimulation of peripheral blood mononuclear cells by this bacterium inhibits the production of inflammatory cytokines (Il-12, interferon gamma) and stimulates the production of IL-10 with an anti-inflammatory role [19,20]. In patients with hereditary risk of IBD, a reduction of *Roseburia* spp. was observed in the intestinal microbiome [19,20].

The above data have been supported by other studies that have shown an increase in the number of species from the Proteobacteria family, especially *Escherichia coli*, in the intestinal mucosa of patients with CD. Among the serotypes of *Escherichia coli*, the one isolated from the intestinal mucosa of adults with CD is adhesion-invasive *E. coli* (AIEC) [21,22]. An increase in the number of AIECs was reported in 38% of patients with CD, compared with 6% of healthy patients [23,24]. These pathogenic bacteria have the ability to adhere to the intestinal epithelium, to alter intestinal permeability, and to induce intestinal inflammation by regulating the expression of inflammatory genes [21,22,23,24]. According to El Ashmar et al., exposure of intestinal epithelium not only to pathogens but also to commensal bacteria could lead to increased intestinal permeability (host-dependent zonulin secretion causes the impairment of the small intestine barrier function after bacterial exposure) [25]. Moreover, Arietta et al. confirmed that this alteration was associated with the development of colitis (reducing low intestinal permeability results in attenuated colitis in the IL10 gene-deficient mouse) [26]. In human models, Caviglia et al. showed that patients with IBD had increased intestinal permeability measured by serum zonulin compared to healthy subjects [27].

Another pathophysiological hypothesis incriminated in the development of IBD is the impairment of the production of metabolites by intestinal dysbiosis. An example is the decrease in the concentration of short-chain fatty acids (SCFAs) secondary to the production of butyrate by *Faecalibacterium prausnitzii* or other species from Clostridium clusters IV, XIVa, or XVIII [18]. The consequence of this decrease in SCFAs is the alteration of regulatory T-cell differentiation and expression, and thus the growth of epithelial cells and the maintenance of intestinal homeostasis [28,29].

**Figure 1:**
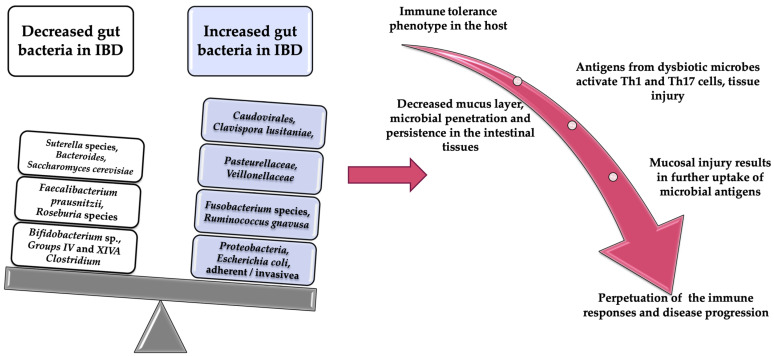
Altered gut bacteria implication in the pathogenesis of IBD.

Besides the bacteria from the Enterobacteriacae family, another class of adherent and invasive bacteria in the mucosa of the digestive tract with a role in the development of IBD are those from the Fusobacteria family [29,30,31,32]. Fusobacterium species mainly colonize the oral cavity and intestine and have been shown to be abundant in the colonic mucosa of patients with UC [32]. An experiment in mice has shown that the administration of *Fusobacterium varium* by rectal enema leads to inflammatory changes in the colonic mucosa. In humans, the bacterium Fusobacterium has been implicated in the pathophysiology of IBD and correlated with the severity of IBD. Another very important role of the Fusobacterium species is its involvement in tumorigenesis. Thus, there are experimental data in mice which claim that these bacterial species are present in greater numbers in a colorectal tumor than in the normal adjacent tissue [33,34]. In the intestinal microflora there are bacteria with a protective role against IBD, such as *Lactobacillus*, *Bifidobacterium*, and *Faecalibacterium*. Their protective role is explained by the stimulation of the production of anti-inflammatory cytokines (like IL-10) and reduction of the production of inflammatory cytokines [35]. Ileal biopsies from CD patients have shown a reduction in the populations of *Faecalibacterium prausnitzii* (which have an anti-inflammatory role), and an increase in the populations of *E. coli* [20,36]. Another bacterium with a possible protective role against the development of IBD has been shown to be *Helicobacter pylori* [20,37]. Altered gut bacteria implication in the pathogenesis of IBD is summarized in Figure 1.

## 3. Probiotics Effects in IBD

Probiotics are microorganisms capable of surviving in the acidic gastric environment. To this point, the existing data support the beneficial role of probiotics in the treatment of IBD [38,39].

The following mechanisms have been suggested for the action of probiotics: stimulation of anti-inflammatory cytokines production (IL-10, transforming growth factor beta (TGF β)), antimicrobial substances secretion, suppression of bacterial growths (thus antimicrobial role), induction of an immune response, immunomodulatory role, improvement of the epithelial barrier function, and suppression of T-cells proliferation [40,41,42,43].

In order for the microorganisms to be considered probiotic agents, they have to meet certain conditions:

- To survive in the pancreatic, biliary, and gastric acidic secretions, and thus to be viable when they reach the small and large intestines;
- To remain viable during transportation and storing;
- To lack toxic or other pathogenic effects on normal human structures;
- To have beneficial effects for the host;
- To adhere to the intestinal epithelial cells;
- To stabilize the intestinal microbiota;
- To produce antimicrobial substances.

Intestinal microbiota have a series of beneficial effects on the normal development of the human organism, but disturbing the homeostasis between the gut bacteria and the immune response can lead to inflammatory changes [44,45,46].

The interaction between probiotic agents and intestinal epithelial cells leads to a decrease in the response of various pro-inflammatory stimuli [47]. This reaction can be explained by the inhibition of the degradation of the nuclear factor kB (IkB/NF-kB) and also by the inhibition of the IkB/NF-kB pathway. Thus, nuclear translocation of NF-kB and the corresponding gene expression are prevented [48].

There is a study that used lining samples from CD patients to observe the existing differences between epithelial cells cultures with no probiotics and those treated with probiotic agents, such as non-pathogen *E. coli* species, *Lactobacillus casei DN-114001*, *Lactobacillus bulgaricus LB10,* and *Lactobacillus crispatus*, after 24 h. The following differences were detected: TNF-alpha release from the epithelial cells was significantly reduced in the cultures treated with *Lactobacillus casei DN-114001* and *Lactobacillus bulgaricus*, in opposition to the ones treated with *Lactobacillus crispatus* and *E. coli*, where no important changes were noticed. In addition, in the cultures treated with *Lactobacillus casei DN-114001* and *Lactobacillus bulgaricus*, besides the TNF-alpha expression reduction in intraepithelial lymphocytes, a decrease of CD4 cells number could be noticed. Probiotics interact with immunocompetent cells by modulating pro-inflammatory cytokines’ local production. The immune system mediators that are responsible for recognizing pathogenic agents are the toll-like receptors (TLRs) [49].

Summarizing the existing information so far, the main immune effects of this microorganism are as follows:

- Reduction of the activity of nuclear factor kappa B (NF-kB);
- Increase of the activity of natural killer cells (NK);
- Involvement in the maturation of dendritic cells;
- Stimulation of cytokines (IL 10) production;
- Activation of antigen presenting cells found in Peyer’s plaques [50,51,52,53,54].

### 3.1. Crohn’s Disease

Most studies that have evaluated the role of probiotic agents in CD have demonstrated their effectiveness in maintaining the clinical remission of the disease.

A study conducted in 2000 showed that *Saccharomyces boulardii* combined with mesalazine reduce the recurrence rates in adults with CD [55]. Another study, conducted in 2013, compared two groups of patients: one group who underwent basic therapy with *Saccharomyces boulardii* and another group who combined this with a placebo [56]. However, this study did not demonstrate significant efficacy of *Saccharomyces boulardii* compared to placebo, in terms of IBD recurrence rate (47.5% vs. 53.2%) [56].

An experiment in mice showed that supplementation with *Saccharomyces boulardii* leads to reduced intestinal permeability and secondary bacterial translocation. However, an immunomodulatory effect of this microorganism has been demonstrated, by increasing plasma levels of IL-10 and intestinal IgA secretion [57].

Another study of patients with CD showed a beneficial effect of using “Synergy 1”, a product containing *Bifidobacterium longum*, oligofructose, and inulin. The administration of this product for a period of six months led to the reduction of proinflammatory biomarkers such as TNF-α in the intestinal mucosa, reduction of the disease activity index, and also improvement of histological parameters [58].

Fedorak et al. evaluated in 2015 the efficacy of another probiotic agent, VSL#3 (containing *Bifidobacterium infantis*, *Bifidobacterium breve*, *Bifidobacterium longum*, *Streptococcus thermophilus*, *Lactobacillus paracasei*, *Lactobacillus acidophilus*, *Lactobacillus recarurus*, in L.) in patients with CD undergoing surgical treatment (intestinal resection). Two groups were formed: the first group consisted of patients with CD who received VSL#3 immediately after surgery and maintained this treatment for a period of 365 days; the second group consisted of patients who received VSL#3 for a period of 275 days (from day 90 after surgery until day 365). Ninety days post-operatively, the two groups—the first of which had already been treated with VSL#3 for 90 days, while group 2 had not yet received VSL#3—underwent endoscopic investigation. However, this evaluation did not show significant differences between the two in terms of intestinal lesions. The second evaluation was performed at 365 days, at the end of the study. According to this evaluation, group 1, who received VSL#3 immediately after surgery, had a lower level of pro-inflammatory cytokines (Il-8 and IL-1b) in the intestinal mucosa and a lower rate of recurrence compared to group 2, in whom VSL#3 treatment was initiated 90 days after surgery. In conclusion, this study proved that the administration of probiotics in patients with CD is more effective if initiated immediately after surgery, compared to late initiation [59].

Two other studies evaluated the role of probiotic agents in inducing remission in patients with CD, demonstrating an improvement in the CDAI score among patients who received these microorganisms. However, these studies used different preparations and observed, in total, only 14 patients. The first study evaluated the efficacy of Lactobacillus and Bifidobacterium. The second study initially included 11 patients who were randomized into two groups: the first group received a placebo agent, and the second group received *Lactobacillus rhamnosus GG* in combination with antibiotics and steroids, over a period of one week. Of these, only five patients reached the end of the study and no additional benefits of *Lactobacillus rhamnosus GG* were shown compared to placebo [60,61]. *Lactobacillus rhamnosus GG* has also been evaluated in children with DCI but has not shown additional benefit over placebo [62,63].

Other studies have evaluated the effectiveness of *Lactobacillus johnsonii* and *E. coli Nissle* in maintaining IBD remission, but without showing significant benefit [64].

Current data suggest that the concomitant use of multiple microorganisms in patients with IBD leads to better results on disease progression than the use of a single microorganism. The most effective have been the microorganisms in the families *Bifidobacterium* and *Saccharomices boulardii*. The determination of the optimal dose represents another problem regarding the use of probiotics in IBD. Many studies use higher than recommended doses, while other studies do not specify the dose used. Two special categories of patients are children and elderly patients, the data on the use of probiotics in IBD in these categories being much more limited. Thus, we need further studies to monitor the effective use of probiotics in IBD among these patients [65].

### 3.2. Ulcerative Colitis

The effectiveness of combining probiotic agents with standard therapies has been evaluated in numerous studies and among patients with UC. One study, which included 244 patients with mild or moderate forms of UC, looked at the benefits of combining *Saccharomices boulardii* and VSL#3 with conventional therapy. According to this study, the products mentioned above did not significantly contribute to the improvement of the remission rates of the disease, but they proved a benefit in reducing disease activity [66].

Nevertheless, other studies have evaluated the effectiveness of VSL#3 in UC, with more promising results. Sood et al. demonstrated that combining VSL#3 with standard therapy over a 12-week period improves disease remission rates by reducing ulcerative colitis disease activity index (UCDAI) score by more than 50%. Another important conclusion was the improvement of lesions from the level of the colonic mucosa at the endoscopic evaluation in the group of patients who used VSL#3 [67].

The effectiveness of VSL#3 has also been proven among children with UC. Thus, a study that followed 29 children with UC over a 1-year period showed a significant improvement in the remission rate of the disease by combining VSL#3 with 5-aminosalicylic acid (5-ASA) and steroids therapy, compared with patients who combined placebo agents with this standard therapy (93% vs. 61%) [68]. Another study that looked at 18 children with UC reported both an improvement in histological scores and a reduction in the level of inflammatory markers by combining VSL#3 with standard therapy [69].

Another probiotic agent that has been shown to be effective in improving endoscopic and histological scores in patients with UC is bifidobacteria-fermented milk (a combination of *Bifidobacterium strains* and *Lactobacillus acidophilus*) [70]. It is worth mentioning that the concentration of SCFAs in feces is increased in the case of the probiotic-treated group vs. a placebo group. However, a more recent study on 195 patients, describing a similar strategy of treatment (which included fermented milk *containing B. breve* + *L. acidophilus*), demonstrated no efficacy to cure or at least to maintain the UC remission [71]. In healthy volunteers, from a practical point of view, *B. bifidum* usage as single-strain-containing probiotic has been shown to be sufficient to enhace SCFAs levels in feces [72]. Taking into account all these data, however, the probiotic’s protective role in both UC and/or CD remains insufficiently known.

Despite some discrepancies regarding the number of patients used in the studies mentioned above, the first study was the only one to confirm the increased number of Bifidobacteria in the feces of probiotics-treated patients and to perform endoscopic analysis.

In the literature there is, however, data suggesting that the use of certain microorganisms may even negatively influence the evolution of patients with IBD. For example, a Danish study comparatively followed two groups of patients with UC over a period of 7 weeks: the first group underwent the standard therapy of *E. coli* with foreign Nissle, and the second group, a placebo agent. The result was that the group of patients taking probiotic therapy had a higher dropout rate and an even lower rate of clinical remission. The hypothesis of the unfavorable effect of *E. coli Nissle* on the evolution of IBD is supported [73]. On the other hand, the rectal administration of Escherichia coli Nissle for proctitis or proctosigmoiditis did not demonstrate additional benefits over placebo [74].

Regarding rectally administered probiotic products, *Lactobacillus reuteri ATCC 55730* in combination with mesalamine has shown benefits in ameliorating mild to moderate forms of UC in children. The study which evaluated this biological compound looked at two groups of pediatric patients with mild or moderate forms of UC; the first group combined *Lactobacillus reuteri ATCC* *55730* with standard mesalamine therapy, and the second group combined placebo with mesalamine. At the end of the study, patients in the first group had both a better clinical response, objectified by reducing the Mayo Disease Activity Index [MDAI] by ≥2 compared to group 2 (100% vs. 53%), and a better remission rate, objectified by an MDAI score of <2.0 (31% vs. 0%) [75].

Among patients with mild to moderate forms of UC, a number of studies have compared the effectiveness of using probiotic agents with standard 5-ASA therapy. The probiotic agents evaluated were *Escherichia coli Nissle 1917*, *Bifidobacterium breve strain Yakult*, *Bifidobacterium breve,* and *Saccharomyces boulardii*. These studies concluded that the probiotic agents mentioned above have a similar efficacy to 5-ASA in maintaining clinical and histological remission in patients with mild to moderate forms of UC [76,77,78,79].

An important therapeutic target in the management of patients with IBD is the prevention of early relapses [80]. Thus, in 2010, a small study, which followed six patients with UC in remission, reported that maintenance treatment with 400 mg rifaximin and 500 mg *Saccharomyces boulardii* led to the maintenance of clinical remission after three months of use. The conclusion was that this therapeutic combination might be useful in preventing early relapses in UC [81].

**Figure 2:**
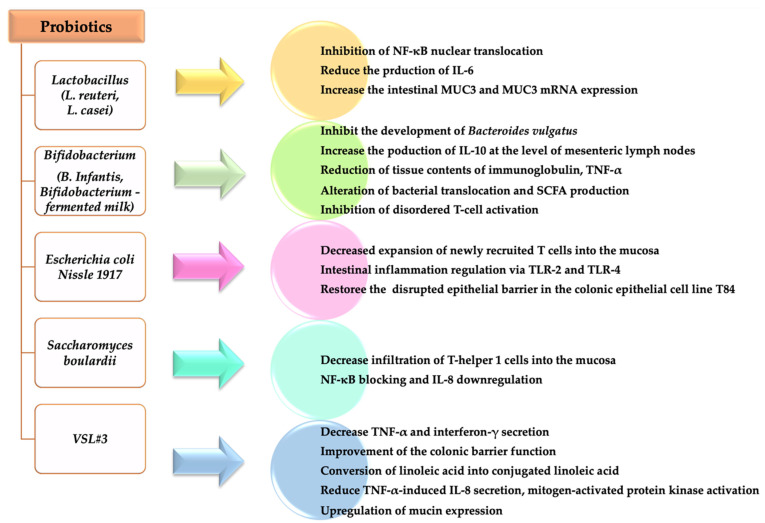
Effects of different probiotics in IBD.

The main effects of different probiotics in IBD are summarized in Figure 2 [79,82,83,84,85]. Dose, treatment duration, and efficiency of probiotic in UC and Crohn’s disease are presented in Table 1.

## 4. Prebiotics

The current therapeutic management of patients with IBD aims, in addition to obtaining clinical and histological remission, to identify alternative methods that lead to the reduction of costs and toxicity involved in immunosuppressive therapies. Prebiotics are non-absorbed carbohydrate polymers, as opposed to probiotics, which are living microorganisms. Prebiotics include fructo-oligosaccharides, inulin, and galacto-oligosaccharides, compounds that stimulate the growth and metabolic activity of beneficial bacteria that are part of the intestinal microflora, such as Bifidobacterium and Lactobacillus species. Another important action of prebiotics is to stimulate the bacterial production of short-chain fatty acids, such as butyrate, with immunoregulatory effects. These effects include histone deacetylase and PPARγ activities with activation of regulatory T cells via G protein receptor 43 and GPR109a and inhibition of pro-inflammatory cytokines [94,95,96]. On the other hand, butyrate is also a metabolic fuel for colon epithelial cells. Numerous experimental studies have demonstrated the effectiveness of oral use of prebiotics alone or in combination with probiotic agents (symbiotics). However, data on the efficacy of these therapeutic preparations in IBD in humans is limited [97,98].

In CD, the use of prebiotics did not show a significant improvement in the evolution of these patients. A study looked at the effectiveness of taking fructo-oligosaccharides in patients with active CD over a period of four weeks. The authors concluded that therapeutic supplementation with this prebiotic agent does not improve the remission rate of CD [99]. However, the combined administration of a prebiotic agent with Bifidobacterium longum in patients with active CD showed both an improvement in clinical and histological scores and a reduction in the concentration of TNFα in the mucosa [100]. In addition, the association of some probiotics with psyllium fiber in steroid refractory CD patients led to 60% remission rates [61]. Another study, however, followed patients with CD who underwent surgical treatment and showed that the combination of four prebiotic agents with four probiotic agents does not bring benefits in preventing postoperative recurrence of this condition [101].

Therapeutic supplementation with oligofructose-enriched inulin does not influence the remission rate of the disease in patients with UC but leads to reduced fecal levels of calprotectin [100]. Inulin is another therapeutic agent that has been shown to improve histological lesions in patients with pouchitis, without improving clinical parameters is [102].

The main action that fructans have on the GI system is the modulation of the intestinal microflora. Numerous studies highlighted the favorable effect of inulin inside the intestine (by acting on the *F. prausnitzii* and *Anaerostipes* sp. levels); this partly explains some of the butyrogenic effects of inulin intake [103,104,105]. It was demonstrated as well that fructo-oligosaccharide (FOS) and galacto-oligosaccharide (GOS) improve the levels of *F. prausnitzii* [106].

In dose–response trials of FOS supplementation, the minimum prebiotics dose capable to induce a bifidogenic effect in healthy subjects was considered 10 g/day, while the dose is decreased (2.5–5 g/day) for inulin-type prebiotics [107,108,109].

Actually, it has been demonstrated that at least two weeks of having vegetables rich in inulin-type fructans in one’s diet results in a 3.8-fold Bifidobacterium genus increase; furthermore, an increase in B. longum subsp. longum was induced (at the species level), and a decrease in the cases of *B. bifidum*, *B. pseudocatenulatum*, and *B. adolescentis* [22,110]. All these data confirm previously published results, which certify that Jerusalem artichoke (which is rich in inulin) intake was corelated to Bifidobacterium increasing [44,47,111,112].

More recent studies have demonstrated that fructans are also able to provide antioxidant actions (higher than in the cases of glucose, fructose, and sucrose) [2], suggesting in this regard that the antioxidant effect is characteristic of FOS. The presence of branches in the molecules and/or the DP influence and induce the antioxidant action of fructans; in this regard, the highest antioxidant-type effects are associated with the branched fructans (agavins) and the linear fructans with a low DP (e.g., inulin Frutafit IQ^®^) [43]. Articles on the topic have proven that the antioxidant capacity of inulin IQ resists during the cooking process, and, respectively, during digestion. Therefore, these observations make even more interesting the rather unstable antioxidant characteristic of fructans (these being known as some of the main water-soluble antioxidants). It is also speculated that inulin-type fructans have the ability to act indirectly, eliminating reactive oxygen species (ROS) (due to the action of SCFA resulting from their fermentation in the colon), and potentiate the activity of antioxidant enzymes glutathione S-transferase (GST) [113].

**Figure 3:**
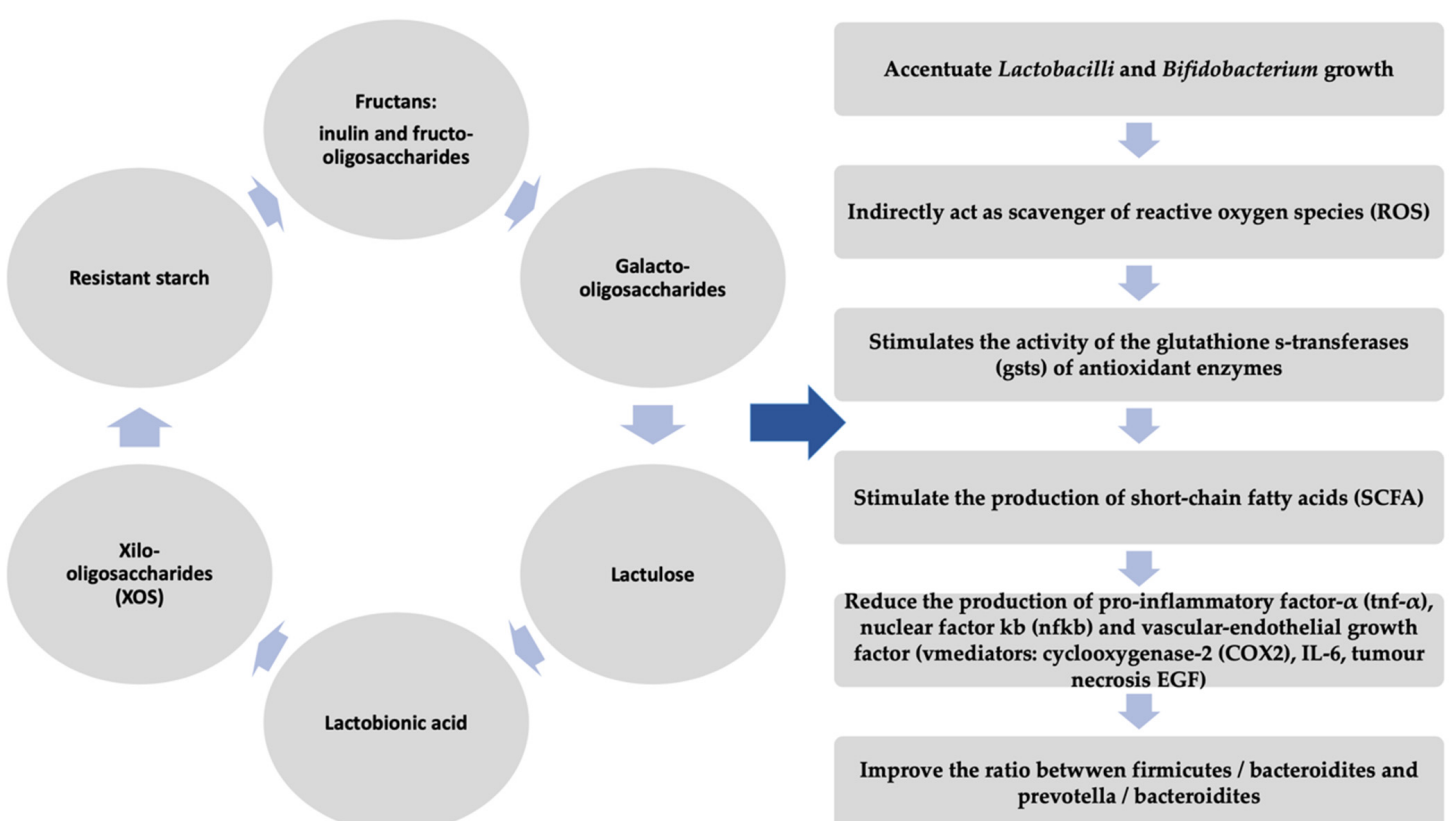
Beneficial effects of prebiotics.

All the studies above demonstrate a beneficial potential of prebiotics (as they are depicted in Figure 3) [114,115,116,117,118,119,120,121,122,123,124,125] in the therapeutic management of IBD, but they have a number of limitations. Thus, future studies are needed to elucidate the pathophysiological mechanisms and therapeutic effects of prebiotics.

## 5. Fecal Microbiota Transplant (FMT)

FMT is currently a potential therapeutic option in patients with a disturbed gut ecosystem. FMT can lead to increased production of short-chain fatty acids, such as butyrate, improving the integrity of the intestinal barrier and thus reducing bowel permeability and severity of IBD. On the other hand, FMT can contribute to the following: the correction of intestinal dysbiosis by inhibiting both T-cell activity and also leukocyte adhesion and the synthesis of pro-inflammatory factors [126]; decreasing colonic inflammation; and initiating intestinal homeostasis restoration by cumulative activation of various immune-mediated pathways [127].

As a procedure, FMT involves harvesting feces from a healthy donor and transplanting them into the patient’s gastrointestinal tract. This therapeutic method has proven to be over 90% effective among patients with recurrent *Clostridium difficile* infection, resistant to standard antibiotic treatment [128,129,130,131]. In 2016, the Food and Drug Administration (FDA) labeled FMT as an Investigational New Drug, and they are now considering it an experimental therapy in IBD [130,131,132].

Preliminary reports of studies that have used FMT in patients with UC or CD have shown promising results regarding the obtaining and maintenance of long-term clinical remission in many cases [133,134]. The profound impairment of the intestinal microbiota among these patients could represent the pathophysiological explanation of the effectiveness of FMT in patients with IBD [133,134].

A recent meta-analysis evaluated 122 patients with IBD who received a FMT (79 with UC, 39 with CD, and 4 with unclassified IBD) and showed a remission rate of 36.2% [135]. The benefits were higher in young patients, especially in the age group 7–20 years. The remission rate was also higher in patients with CD compared to patients with UC (60.5% vs. 22%) [135]. Equivocal results were obtained in other studies that followed a number of RCTs conducted to quantify the FMT efficacy in UC alone [136,137,138].

There have been a number of other studies conducted in both adults and children with IBD with controversial results. For example, one study looked at the effectiveness of FMT administered via the nasojejunal tube in patients with moderate or severe forms of UC. After 12 weeks of administration, only one patient showed an improvement in the Mayo Score [139]. Suskind et al. looked at the effectiveness of administering a single FMT via nasogastric tube to four children. Their study did not observe any clinical benefit of FMT [140]. However, Kunde et al. demonstrated significant benefits of FMT when administered via enema. They monitored nine children with UC who received FMT via enema, in one session per day, for five consecutive days [141]. Another study that included 15 adults with steroid-dependent UC showed benefits of performing FMT via upper endoscopy. Of these, eight patients (57%) showed clinical improvement, and four patients remained in remission for a long time [142].

FMT has been shown to be effective in CD in a number of studies. The increase in the diversity of the intestinal microbiota represents the pathophysiological hypothesis of the benefits of FMT among patients with CD. He et al. followed the evolution of 25 patients with CD and an inflammatory mass after performing a FMT. Of these, 13 (52%) obtained clinical remission three months after the FMT. These patients were subsequently evaluated at 6, 12, and 18 months, with the following results: maintenance of clinical remission was reported in 12 of the patients (52%) at 6 months, in 8 of the patients (32%) at 12 months, and in 5 of the patients (22.7%) at 18 months. Cui et al. also reported an improvement in the clinical remission rates of CD patients who underwent FMT. They followed 30 patients with refractory CD. One month after FMT, 86.7% of patients showed clinical response, with a remission rate of 76.7% [143]. Previous published studies reported that another concern related to FMT is considered the risk of an IBD flare (up to 25% with flares), but recent results suggest a much lower risk [144,145].

Although the results of studies to date are variable, FMT may have benefits in the evolution of patients with IBD. The main pathophysiological effect of this therapeutic method in patients with IBD is the amelioration of intestinal dysbiosis. At present, however, it is not known whether intestinal dysbiosis is a primary or secondary event in the evolution of IBD, and, consequently, the impact of dysbiosis correction on the disease remains unclear. Another concern with FMT is the risk of an IBD flare. Early studies reported up to 25% with flares; however recent data suggest a much lower risk. However, it has been observed that patients who underwent FMT showed a clinical and endoscopic improvement compared to those treated with placebo, suggesting the therapeutic potential of this method [146,147].

## 6. Natural Chemical Compounds in IBD Treatment

Dietary polyphenols are recognized as natural-origin chemical compounds that are included in food products like cereals, fruits [148], vegetables, wine [149,150,151], dark chocolate (cocoa powder), tea and coffee, and so on. From the chemical point of view, these substances are considered an extended heterogeneous group of compounds, having structural units (phenolic or hydroxylated aromatic rings) in common, in all the phenolic derivatives. Phenolic compounds are classified taking into account the number of phenolic rings they contain in their structure, as well as the structural connecting elements between these rings. The main classes of polyphenols (considered dietary) can be listed as follows: phenolic acids, flavonoids, stilbenes, tannins, and diferuloylmethanes [152,153]—their actions and effects on human health (through the diet) being induced by their chemical structure [154].

Phenolic acids contain a carboxylic function linked to a phenol ring, which is classified in benzoic acid derivatives (ie gallic or protocatechuic acids) and cinnamic acid derivatives (i.e., caffeic, p-coumaric, and ferulic acids) [149].

Stilbenes are organic compounds of hydrocarbons, having in their molecule a trans-ethene double bond, which is replaced by a phenyl group (on both atoms of carbon linked by the double bond) [67]. They can be found in lower quantity in plants, more abundant in the form of resveratrol and its isomer (trans-resveratrol), and more frequently in grapes and wine [155]. Flavonoids are represented by >10,000 natural chemical compounds, present in about 9000 species of plants [152,153,156], encompassing a wide diversity in their structures; however, they are derived from a common biosynthetic pathway—that is, the phenylpropanoid metabolic pathway, which comprises of the acetate-malonate and shikimic acid pathway precursors—and lead, for example, to kaempferol, quercetin, luteolin, (epi)catechin, (epi)gallocatechin, and so on.

Tannins are defined as polyphenolic organic compounds with the property of precipitating the proteins (especially the salivary proteins) and providing them an astringent character. These characteristics may explain the tannins’ protective role (against pathogens and herbivores) in plants. From the point of view of physical properties, these polyphenols with high molecular weight (between 500–3000 Da) are water-soluble, being classified in two groups: proanthocyanidins (or condensed tannins which are polymers of flavan-3-ol units) and hydrolysable tannins (which are esters formed between phenolic acids and a molecule of cyclic polyalcohol, frequently glucose).

A group of phenolic compounds, Diferuloylmethanes is a class that includes curcumin, which is a commonly known compound, derived mainly from the turmeric spice [156].

Phytochemicals include an extensive variety of natural compounds derived from plants, and have been reported to exert numerous benefits in several chronic disorders, as per multiple pieces of evidence [157]. The gut microbiota and immunity of the intestine has been reported to be influenced by a number of polyphenols, such as flavonoids, phenolic acids, stilbenes, and lignans, which have numerous biological benefits (antioxidant, anti-cancer, anti-inflammatory, etc.) also presented by hydrophobic polyphenolic derivative of *Curcuma longa* (i.e., curcumin) [158]. The production of cytokines by macrophages and epithelial cells of the intestine is suppressed by blockage of activation of NF-Κb, therefore abbreviating colitis induced by DSS- and TNBS [159,160,161,162].

The polyphenolic compounds present in the diet exhibit limited bioavailability profiles, due to inefficient intestinal absorption of curcumin [162], following which, a large amount of unabsorbed polyphenolic compounds are transported to the large intestine, resulting in their interaction with the gut microbiome of the colon, which further possesses the ability to catabolize polyphenols and degrade them into small fragments [163].

The *E. coli*-derived curcumin-converting enzyme aids the conversion of curcumin into tetrahydro curcumin [164], which prevent colitis and related colon cancer in mice. A significant involvement of polyphenols in CD has been reported by recent epidemiological study. Furthermore, supplements of curcumin have been reported to exert effective outcomes in the induction and maintenance of remission in UC patients, as per clinical studies [165,166,167,168].

However, a very low number of clinical investigations which have directly targeted IBD are available with respect to polyphenol intervention. The investigations, on a general basis, have administered approximately 2 g/d red wine, curcumin, apple, cacao, pycnogenol, and blueberries to the subjects (10 per group), for a period from four weeks to two years. Unfortunately, there are only a very limited number of human trials available that have focused directly on IBD with respect to polyphenol intervention. Chiba et al. conducted a clinical study with 22 CD subjects, and reported maintenance of remission for over two years, by a plant-food-rich semi-vegetarian diet, which was therefore high in polyphenols, unlike an omnivorous diet [169].

Hanai et al. incorporated 2 g of curcumin plus medication per day for over six months to 89 UC patients in a randomized, double-blind, placebo-controlled multicenter intervention study, resulting in potential improvement in UC-associated morbidity parameters (endoscopic and clinical activity index) and recurrence rate [167]. Additionally, besides exerting direct effects, curcumin was also reported to elevate the bioavailability profile of the prescribed medication, via interaction with multiple efflux pumps and/or altered metabolism of phase I/phase II reactions [170].

Koláček et al. conducted a study to investigate the consequences of pycnogenol (i.e., a polyphenolic compound derived from the bark of maritime pine (Pinus pinaster)), comprising procyanidins (70%) at 2 mg/kg BW, on 15 CD patients in remission, for over 10 weeks, where the results were compared to 15 healthy control subjects. However, an accurate comparison was not permitted between the two groups, as the controls were not treated. Elevated concentration of Cu/Zn superoxide dismutase (SOD) was reported in CD patients, as well as enhanced oxidative damage of proteins, unlike in healthy subjects. Inflammatory markers, like calprotectin (i.e., a systemic inflammation-associated protein produced by neutrophils) and CRP were negatively related to the total plasma antioxidant activity (TAC). Following intervention, no significant alteration was reported in most parameters, like F2-isoprostanes, CRP, and reduced glutathione (GSH), as compared to before intervention, whereas levels of lipo-peroxide AOPP were greatly retarded, along with significant elevation in SOD, following intervention. Therefore, unlike inflammatory markers, a significant reduction in oxidative stress markers was reported, making this the first study to investigate the direct actions of hydrophilic polyphenols on IBD patients [171].

Short-term intervention studies have also been conducted, although with rather more contentious results, in part as possible alterations in the inflammation processes during short-term trials. However, in a placebo-controlled study, pain and discomfort in the stomach was abbreviated in children with gastroenterological irritation administered a single dose of a novel prebiotic based upon polyphenols (i.e., 2 ounces of Preliva (Goodgut INC, USA)) rich in Japanese honeysuckle, grape, and pomegranate, unlike the placebo group, without measurement of bacterial cultures and dosage reporting. Furthermore, there is a requirement for more such investigations, preferably with mid-to-long-term polyphenol administration [172].

## 7. Conclusions

In recent years, the importance of intestinal microflora in maintaining the normal functioning of the body has been more and more recognized. Numerous studies have looked at the potential therapeutic effects of the microorganisms that make up the intestinal microbiome in various diseases.

In IBD, therapies based on the intestinal microbiota have demonstrated great therapeutic potential. According to existing data, the benefits of these therapies can be mainly explained by the immunomodulatory effect. Compared to immunosuppressive treatments, the toxicity is much lower. The aim is to identify therapies based on the intestinal microbiome that can be used not only as adjuvants of immunosuppressive therapies, but also as therapies themselves. Moreover, there are data that suggest the potential of probiotics to reduce the risk of developing the disease among high-risk patients, such as those with a hereditary history of IBD [146].

The use of probiotic agents, especially those of the *Lactobacillus* and *Bifidobacterium* species, were reported to be effective in UC, which is why it represents an adjunctive treatment among these patients. Both the improvement of clinical signs and symptoms and the induction/maintenance of the remission period are the main proven benefits of probiotic agents in patients with UC. The mechanisms behind these results are the immunomodulatory and anti-inflammatory effects of probiotic agents. In other words, these products reduce the synthesis of pro-inflammatory mediators and increase the synthesis of anti-inflammatory mediators. Although these effects are potentially present in patients with CD, there are still insufficient data to support the use of probiotics as an adjunctive treatment in patients with CD [65].

## Figures and Tables

**Table 1:** Dose, duration of treatment, and efficiency of probiotic in ulcerative colitis and Crohn’s disease.

Probiotic	Ulcerative Colitis	Crohn’s Disease	Ref.
	Dose of Probiotic; Duration of Treatment; Probiotic Effect		
*Escherichia coli*	25 × 10^9^ CFU/100 mg; 12 weeks; remission period similar with groups treated with standard therapy	10^9^ CFU/day; 1 year; improvement of drug adherence and reduction of recurrence rate	[77,86]
*Escherichia coli* NISSLE 1917.	10^8^/mL—10, 20, 40 mL; 2 weeks; improvement of macroscopic and microscopic parameters	-	[74]
*Saccharomyces boulardii*	250 mg × 3/day; 4 weeks; improvement of remission rate	1 g/day; 6 months; improvement of remission rate	[55,87]
VSL#3 *Bifidobacterium infantis*, *Bifidobacterium breve*, *Bifidobacterium longum*, *Streptococcus thermophilus*, *Lactobacillus casei*, *Lactobacillus plantarum*, *Lactobacillus acidophilus*, and *Lactobacillus delbrueckii*	450–18 × 10^9^ CFU/day; 1 year; improvement of remission rate; improvement of macroscopic and microscopic parameters	9 × 10^11^ CFU × 2/day; 1 year; improvement of remission rate and reduction of intestinal pro-inflammatory cytokines	[59,68]
*Bifidobacterium longum* (BL) and Inulin-oligofructose (IOf)	2 × 10^11^ CFU BL + 6 g IOf × 2/day; 4 weeks; reduction of inflammatory markers	-	[88]
*Bifidobacterium longum*	2–3 × 10^11^ CFU/day; 8 weeks; improvement of remission rate	2 × 10^11^ CFU/day; 6 months; reduction of intestinal pro-inflammatory cytokines, improvement of microscopic parameters, improvement of disease activity index	[89]
*Bifidobacterium breve* (BB) and galacto-oligosaccharide (GoS)	10^9^ CFU × 3/day BB + 5.5 g/day GoS; 1 year; reduction of fecal pH, fecal *Bacteroidaceae,* and myeloperoxidase	-	[90]
*Bifidobacterium breve* (BB), *Bifidobacterium longum* (BL), *Lactobacillus casei* (LC), *Psyllium* (P)	-	30 × 10^9^ CFU/day BB, 15 × 10^9^ CFU/day BL, 30 × 10^9^ CFU/day LC 9.9 g/day P; 8.5–18 weeks; improvement of clinical parameters	[61]
*Lactobacillus rhamnosus* GG	18 × 10^9^ CFU/day; 12 months; improvement of remission rate	10^10^ CFU × 2/day; 6 months; improvement of disease activity index, reduction of intestinal permeability	[60,91]
*Lactobacillus delbruekii* and *Lactobacillus fermentatum*	10 × 10^9^/day; 8 weks; improvement of macroscopic and microscopic parameters	-	[92]
*Lactobacillus salivarius*, *Lactobacillus acidophilus* and *Bifidobacterium strain BGN4*	Unspecified; 2 years; improvement of disease activity index and of remission rate	-	[93]

Legend: CFU—colony-forming units.
